# Investigating the Potential of CO_2_ Nanobubble Systems for Enhanced Oil Recovery in Extra-Low-Permeability Reservoirs

**DOI:** 10.3390/nano14151280

**Published:** 2024-07-30

**Authors:** Liyuan Cai, Jingchun Wu, Miaoxin Zhang, Keliang Wang, Bo Li, Xin Yu, Yangyang Hou, Yang Zhao

**Affiliations:** Key Laboratory for EOR Technology (Ministry of Education), Northeast Petroleum University, Daqing 163318, China; webmaster@nepu.edu.cn (L.C.); sygc8810@163.com (M.Z.); wangkl0608@126.com (K.W.); nepu_li@163.com (B.L.); 13993426757@163.com (X.Y.); hyy1912982482@163.com (Y.H.); dqzy0683@163.com (Y.Z.)

**Keywords:** nanobubble, CO_2_-EOR, channeling prevention, nanoparticle, stability

## Abstract

Carbon Capture, Utilization, and Storage (CCUS) stands as one of the effective means to reduce carbon emissions and serves as a crucial technical pillar for achieving experimental carbon neutrality. CO_2_-enhanced oil recovery (CO_2_-EOR) represents the foremost method for CO_2_ utilization. CO_2_-EOR represents a favorable technical means of efficiently developing extra-low-permeability reservoirs. Nevertheless, the process known as the direct injection of CO_2_ is highly susceptible to gas scrambling, which reduces the exposure time and contact area between CO_2_ and the extra-low-permeability oil matrix, making it challenging to utilize CO_2_ molecular diffusion effectively. In this paper, a comprehensive study involving the application of a CO_2_ nanobubble system in extra-low-permeability reservoirs is presented. A modified nano-SiO_2_ particle with pro-CO_2_ properties was designed using the Pickering emulsion template method and employed as a CO_2_ nanobubble stabilizer. The suitability of the CO_2_ nanobubbles for use in extra-low-permeability reservoirs was evaluated in terms of their temperature resistance, oil resistance, dimensional stability, interfacial properties, and wetting-reversal properties. The enhanced oil recovery (EOR) effect of the CO_2_ nanobubble system was evaluated through core experiments. The results indicate that the CO_2_ nanobubble system can suppress the phenomena of channeling and gravity overlap in the formation. Additionally, the system can alter the wettability, thereby improving interfacial activity. Furthermore, the system can reduce the interfacial tension, thus expanding the wave efficiency of the repellent phase fluids. The system can also improve the ability of CO_2_ to displace the crude oil or water in the pore space. The CO_2_ nanobubble system can take advantage of its size and high mass transfer efficiency, among other advantages. Injection of the gas into the extra-low-permeability reservoir can be used to block high-gas-capacity channels. The injected gas is forced to enter the low-permeability layer or matrix, with the results of core simulation experiments indicating a recovery rate of 66.28%. Nanobubble technology, the subject of this paper, has significant practical implications for enhancing the efficiency of CO_2_-EOR and geologic sequestration, as well as providing an environmentally friendly method as part of larger CCUS-EOR.

## 1. Introduction

Extra-low-permeability reservoirs are characterized by the presence of micro- and nanopores, which serve as the primary storage space for crude oil. These reservoirs exhibit strong capillary forces [1]. The main production enhancement measures employed to improve the recovery rate of extra-low-permeability reservoirs include CO_2_ injection, CO_2_ throughput, low-salinity water injection, and the spontaneous seepage of surfactant [2]. The rapid diffusion of directly injected carbon dioxide along the fracture reduces the contact time and contact area of carbon dioxide with the ultra-low-permeability petroleum matrix, thereby hindering the effective action of carbon dioxide molecular diffusion [3]. Due to the difference in fluidity between CO_2_ and crude oil, especially the influence of reservoir non-homogeneity, it is very easy for CO_2_ injected into tight reservoirs to escape along the dominant channels (cracks); therefore, CO_2_ channeling prevention is the key to realizing CO_2_ burial and efficient drainage and expulsion. At present, the main blocking technology used involves the blocking of the hypertonic layer by injecting different blocking agents, which mainly include foam, gel, ethylenediamine salt, lime water, and so forth [4]. Due to the half-life of the foam in the formation, this technology will not pollute the formation, and it is one of the most commonly used plugging technologies at this stage [5]. Nanobubbles with ultra-long-life bubbles have a small particle size and large specific surface area, which enable them to exhibit ultra-high dissolution and mass transfer efficiency and ultra-long life. These properties make them an excellent medium to promote spontaneous imbibition [6]. Consequently, the construction of a gas–liquid dispersion system, comprising CO_2_ as the dispersed phase, low-salinity water and surfactant solution as the continuous phase, and bubble sizes in the micro- and nanoscales, represents a potentially powerful candidate for promoting the efficient development of extra-low-permeability oil [7]. However, there are still numerous scientific challenges that need to be overcome to facilitate the promotion and application of CO_2_ nano-dispersion systems [8]. As nanopores represent the primary storage space for crude oil in extra-low-permeability reservoirs, the injectivity and stability of the CO_2_ nano-dispersion system are essential prerequisites for determining the suitability of the system for extra-low-permeability reservoir development [9]. Further clarification regarding the gas–liquid dispersion system with a bubble size on the order of nanometers is required concerning the basic characteristics (size, size distribution, and bubble number concentration) and the stability of CO_2_ nanobubbles under static and flow conditions [10]. Such findings will provide a reference for the promotion of the application of this system in the development of extra-low-permeability reservoirs.

Nanobubbles are gas–liquid dispersed systems with dimensions on the order of nanometers [11]. They are formed by the dispersion of gases in liquids, and they possess a number of unique properties, including small bubble size, a large specific surface area, a long retention time in the bulk phase, high stability, and high solvation and mass transfer efficiencies [12]. Nanobubbles can be classified into surfactant-free nanobubbles and surfactant-containing nanobubbles. Surfactant-free nanobubbles are mainly composed of gas and liquid phases, and the physicochemical properties of the gas are the main factors that affect their performance [13]. Depending on the application requirements, common gases, such as air, CO_2_, and N_2_, can be used as the inner core of the bubbles, and the liquid–phase composition is usually dominated by mixtures consisting of deionized water or inorganic salt solutions. Surfactant nanobubbles usually consist of a compressible gas core, a shell layer, and a liquid phase, with the gas type being similar to the composition of micro- and nanobubbles [14]. The liquid phase is mainly used to form an ultra-low-permeability molecular layer at the periphery of the gas core by adding surfactants and polymers to slow down the diffusion of gases and prolong the lifetime of the nanobubbles [15]. Nanobubbles with small size and high specific surface area help to improve the mass transfer rate at the gas–liquid interface and promote chemical reactions and physical adsorption [16]. The small radius of curvature and high internal pressure of nanobubbles increase the solubility of gases (e.g., CO_2_ and N_2_) in the liquid phase. These properties have attracted attention in solving many industrial frontier problems. They are widely used in wastewater treatment, the removal of pollutants from sediments and soils, and other environmental and biomedical aspects [17,18,19,20,21].

The current mainstream theory attributes the stable existence of nanobubbles to the charged gas–liquid interface, which generates an electrostatic repulsive force that blocks the aggregation of bubbles, so that the internal additional pressure is balanced by the external electrostatic repulsive force, leading to their stable existence [22,23,24]. The hydrophobic nanoparticle surface facilitates nanobubble nucleation and improves suspension stability. Nanobubbles can modify the surface properties of nanoparticles through mechanisms such as changing the nanoparticle surface charge, promoting nanobubble nucleation, and interacting with nanoparticles of different charges [25]. In addition, the presence of a large number of nanoparticles and impurities in the solution can lead to the adsorption of nanomaterials at the bubble interface and the formation of armored nanobubbles, and the presence of a shell layer around the bubbles increases the stability of the nanobubbles by reducing the gas diffusion rate and counteracting the Laplace pressure [26].

Given the low porosity of extra-low-permeability oil reservoirs, it is anticipated that the capacity for extra-low-permeability oil production could be significantly enhanced by dispersing gases in liquids to form a stable nanoscale dispersion system utilizing the distinctive properties of nanobubbles. The injection of CO_2_ in the form of nanobubbles into underground saline formations or oil and gas reservoirs has a number of advantages: (1) The small size and low buoyancy of nanobubbles reduce the capillary force of fluids entering the rock pores. In addition, nanobubbles can inhibit the occurrence of scuttling and gravitational overcovering of CO_2_ in the formation, thus increasing the wave efficiency of the replacement phase fluid and improving the ability of CO_2_ to displace crude oil or water in the pore space [27,28,29]. (2) The high internal pressure of nanobubbles significantly increases the dissolution and mass transfer rate of CO_2_ in water or oil, which is conducive to increasing the dissolution rate and solubility of CO_2_ in the aqueous or oily phase [30,31,32,33]. (3) In addition, micro- and nanobubbles are characterized by simple and low-cost preparation methods, which can significantly reduce the cost of enhanced recovery while simultaneously avoiding the contamination and destruction of underground reservoirs by chemicals [34,35,36]. In CO_2_-EOR and geological storage applications, nanobubbles have shown potential application value as suitable gas carriers. Therefore, a systematic study of the fundamental properties and stability mechanisms of nanobubbles and their potential application value in CO_2_-EOR and geological storage is needed. First, nanobubble technology is of great practical importance to improve the efficiency of CO_2_-EOR and geological storage. Conversely, it will provide a novel, economical, and environmentally friendly method for CO_2_-EOR and geological storage [37,38]. On the other hand, it will provide a novel, economical, and environmentally friendly method for CO_2_-EOR and geological storage [39,40].

In this paper, a modified nano-SiO_2_ particle with pro-CO_2_ properties was designed using the Pickering emulsion template method and employed as a CO_2_ nanobubble stabilizer. The initial preparation of the CO_2_ nanobubble system was conducted using a commercially available micro- and nanobubble generator. In the subsequent investigation, the effects of surfactant type, temperature, and oil content on the formation and stability of CO_2_ nanobubbles were examined under high temperatures and pressure and were investigated by utilizing a high-temperature and high-pressure visualization device. The wettability, interfacial properties of the CO_2_ nano-dispersion system and size stability were investigated. The displacement effect and recovery enhancement mechanism of the CO_2_ nanobubble dispersion system were investigated through a series of core flooding experiments.

## 2. Materials and Methods

### 2.1. Materials

The following chemicals were used in the studies: silicon dioxide nanoparticles (SiO_2_, purity of 99.5%, a melting point of 1610 °C, and a packing density of 100 kg/m^3^) were provided by Aladdin Biochemical Technology Co., Shanghai, China. The supplier provided silica nanoparticles with an average particle size of 20–50 nm. Paraffin wax (melting point 45–58 °C), trichloromethane (99.9% purity), sodium chloride (98.5% purity), sodium bicarbonate (98.9% purity), calcium chloride (99.9% purity), potassium chloride (99.9% purity), sodium bicarbonate (99.9% purity), sodium hydroxide (99.9% purity), magnesium chloride (purity 99.9%), dimethicone (viscosity of 50 cs and a flash point of 318 °C), the anion–nonionic surfactant sodium dodecyl sulfate (SDS, 99.9% purity), the cationic surfactant hexadecyl trimethyl ammonium bromide (CTAB, 99.9% purity), and the anion–nonionic surfactant JNPs-3 (99.9% purity) were developed in a laboratory. All standards were purchased by Aladdin Biochemical Technology Co., Shanghai, China. The oil used in the study was provided by Daqing Oilfield (Daqing, China).

### 2.2. Preparation and Characterization of Modified Nanoparticles

**Figure 1:**
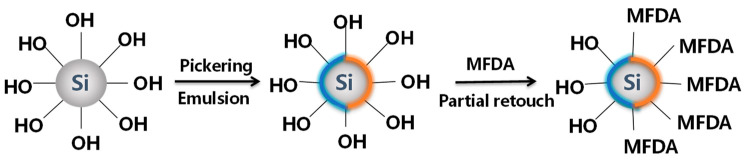
Janus functional particle synthesis program.

**Figure 2:**
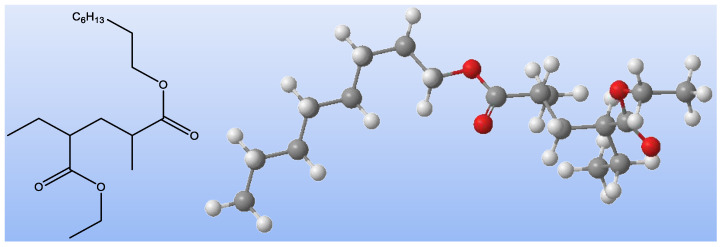
MFDA molecular formula.

The following work is based on the Pickering emulsion template method, and the synthesis scheme is shown in Figure 1. First, 0.5 g of SiO_2_ nanoparticles was dispersed with 3 g of solid paraffin wax in 200 mL of double-distilled water at a temperature of 65 °C. Emulsification was carried out using a high-speed mixer (EU50, FLUKO, Shanghai, China) at 2000 rpm. The mixture was emulsified using a high-speed blender for 3 h. Immediately after emulsification, the mixture was transferred to a refrigerator for 4 h to solidify the paraffin. The non-adsorbed SiO_2_ particles on the surface were washed off with a specific amount of double-distilled water, and the hardened paraffin emulsion was vacuum-dried at 45 °C. The paraffin emulsion droplets at the end of the drying process were dispersed in an ethanol solution of 100 mL of CO_2_-philic hydrophobic polymer monomer (MFDA) at a concentration of 0.2% and reacted at room temperature for 72 h. At the end of the reaction, the paraffin emulsion droplets were filtered and rinsed with anhydrous ethanol to remove the unreacted MFDA and the nano-SiO_2_ particles. The paraffin was then dissolved with trichloromethane, rinsed via centrifugation, collected to release the modified SiO_2_ particles, and vacuum-dried for 24 h. The molecular structure of the CO_2_-philic hydrophobic polymer monomer (MFDA), as shown in Figure 2, was grafted onto part of the surface of the SiO_2_ particles to prepare SiO_2_@ MFDA Janus particles on one side of the hydrophilic, negatively charged silicone hydroxyl (Si-OH) after ionization and on the other side of the hydrophobic, temperature-resistant hydrophobic polymer monomer, named Smart Nano-29.

In the study presented herein, we used four techniques, namely, the use of a laser particle size analyzer (Zetasizer NanoZs90, Malvern Panalytical, UK), infrared spectroscopy (Nicolet iS20 FTIR, Thermo Fisher Scientific, Waltham, MA, USA), transmission electron microscopy (Talos F200i S TEM, Thermo Fisher Scientific, USA), and thermo-gravimetric analysis (TGA 4000, PerkinElmer, Waltham, MA, USA), to characterize the Smart Nano-29 nanoparticles.

### 2.3. CO_2_ Nanobubble System Preparation

**Figure 3:**
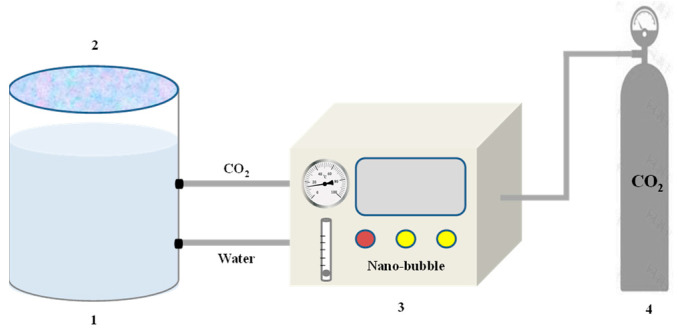
Schematic diagram of CO_2_ nanobubble preparation process. 1—CO_2_ nanobubbles; 2–Masking plate; 3–Nanobubbles generator; 4—CO_2_ cylinder.

Prior to the commencement of the experiment, the apparatus and beaker container were rinsed three to five times with deionized water and ethanol. A plastic baffle was used to cover the top of the glass beaker throughout the experimental process, ensuring that the CO_2_ nanobubbles generated during the preparation and testing process were not contaminated by other substances. The CO_2_ nanobubble preparation process is schematically shown in Figure 3. Three surfactant solutions (SDS, JNPs-3, and CTAB) with a concentration of 0.15 wt.% were prepared using deionized water. Smart Nano-29 with a concentration of 0.05 wt.% was then added to the surfactant solution, which was fully dispersed using an ultrasonic disperser. In order to gain further insight into the fundamental properties of carbon dioxide nanobubbles, our testing was undertaken using high-pressure CO_2_ lines (approximately 50 psi), and our gas connections were connected to the nanobubble generator via an in-line mechanical pressure gauge using Ohmeda valves and the nanobubble generator, which was then linked to a glass beaker with a capacity of 2 L. The control panel of the nanobubble generator allows the user to set the gas–liquid cycle time (0–100 min) and adjust the gas flow rate (0–200 mL/min).

### 2.4. CO_2_ Nanobubble System Number Concentration

The experimental methodology for quantifying the number concentration of carbon dioxide nanobubble systems is as follows: firstly, the particle size distribution of a standard silica nanoparticle suspension dispersed in deionized water of known concentration must be measured in order to establish a correlation between the concentration of a stable silica suspension and the average count rate. The average count rate (ACR) values obtained when the nanobubbles were measured using a dynamic light scattering (DLS) test employing a laser particle sizer (Zetasizer Nano, Malvern Instruments, Malvern, Worcestershire, UK) were employed to quantify the number concentration of nanobubbles. The intensity signal was collected using non-invasive backscatter detection at an angle of 173°. A 1 mL sample was taken from the original cuvette and measured after approximately five minutes. The measurements were conducted with an automatic attenuator at 25 °C. The refractive index of the solvent was set to 1.33 at 25 °C. Based on the detected scattering intensity signals, the size distribution, the mean diameter, and the polydispersity index were calculated using an autocorrelation function. Each sample was tested at least six times. To obtain the concentration of CO_2_ nanobubbles using the nanoparticle tracking analysis (NTA) system, a clear video of the Brownian motion of the CO_2_ nanobubbles in the field of view, which appears as a scattered light spot with a diffraction pattern, was first taken. Next, the CO_2_ nanobubbles in each frame were identified using the “Find Maximum” method in Image J (v1.8.0.112). The CO_2_ nanobubbles in each frame were identified using a Gaussian fit with sub-pixel accuracy. The mean and standard deviation of the numerical concentrations were calculated from the data of 15 random images for each test. The concentration of CO_2_ nanobubbles in the solution should not be too high to avoid interactions between CO_2_ nanobubbles as much as possible. After identifying individual CO_2_ nanobubbles in each frame, it is necessary to connect the positions in a bunch of image sequences, which can be achieved by the Nano Track J (v1.0.3) [41] plugin in Image J. This method allows a semi-quantitative study of the concentration of the number of CO_2_ nanobubbles produced.

### 2.5. CO_2_ Nanobubble System Stability

**Figure 4:**
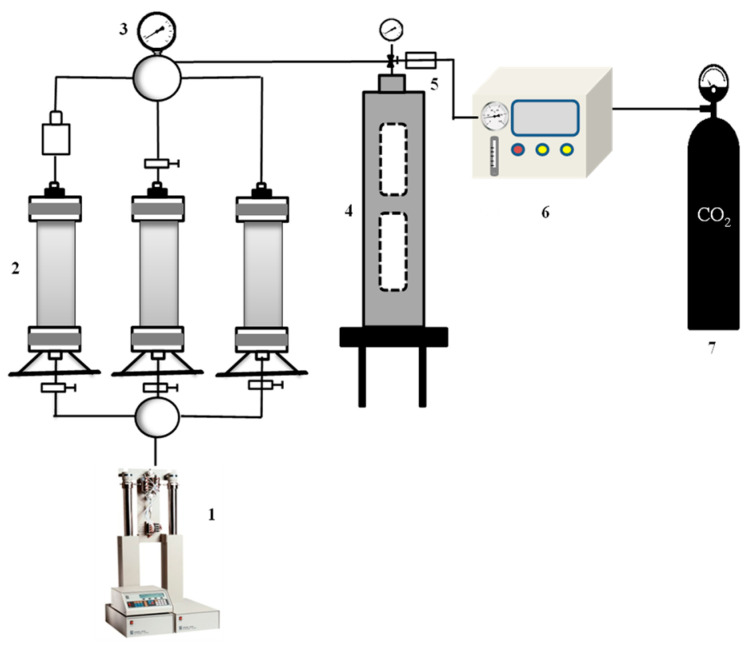
High-temperature and high-pressure visualization foam analyzer. 1—ISCO high-pressure piston pump; 2—Intermediate vessel; 3—Precision pressure gauge; 4—High-temperature and high-pressure visualization reactor; 5—Return valve; 6—Nanobubbles generator; 7—Gas cylinder.

The effects of surfactant type, temperature, and oil content on the stability of CO_2_ nanobubbles were investigated. The structure of the high-temperature and high-pressure visual foam analyzer (manufactured by MGA Technologies Co., Moirans, French) used in the experiment is shown in Figure 4. Three different types of surfactants (SDS, JNPs-3, and CTAB) at a concentration of 0.15 wt%were pre-prepared in 200 mL of simulated stratum water and stirred in a magnetic stirrer for 5–10 min. Subsequently, 200 mL of the surfactant solution was added to a high-temperature and high-pressure reactor, which was connected to a constant-temperature oil bath. A specified amount of dimethyl silicone oil was added to the oil bath, and the experimental temperature was regulated using an oil bath heating device to measure the foaming performance under different temperature conditions. Crude oil with different contents was added to the surfactant solution, and the foam properties were evaluated under different oil content conditions. We set the gas mass flow rate and the power supply of the flow indicator to the on state, waited for the zero point to stabilize, and then carried out ventilation. A flow rate of 100 mL/min was maintained for 12 s prior to commencing the measurement procedure. At the conclusion of the measurement period, the foaming volume (V_max_) and half-life (t_1/2_) were recorded, after which the Foam Composite Index (FCI) was calculated. The foaming agent in the jacketed cylinder was then poured into the waste pool, and the interior was cleaned.

### 2.6. CO_2_ Nanobubble System Wettability

Using an optical contact angle measuring instrument (OCA25, DataPhysics Instruments, Baden-Württember, Germany), the contact angle of the CO_2_ nanobubble system was quantified using the hanging drop method to evaluate changes in the system’s wet stability. A thin slice of the core sample from the target reservoir was placed on the test platform of the contact angle tester. The position of the camera was adjusted so that the thin slice could be clearly presented in the field of view. A drop of water was dropped with a syringe, and the image was photographed. Relevant software was then used to determine the contact angle of the water droplet on the thin slice.

### 2.7. CO_2_ Nanobubble System Interface Features

In the present study, the surface tension of CO_2_ nanobubble systems formed from different solutions was quantified utilizing a DCAT25 surface tension meter manufactured by DataPhysics, Germany. The variation in the surface tension values of CO_2_ nanobubbles in different solutions was tested. The surface tension values of CO_2_ nanobubble systems formed by deionized water, Na^+^ solution, Ca^2+^ solution, and 0.15 wt%surfactant solution were measured from 0 to 60 min in turn. Surface tension was determined by the pendant drop method.

### 2.8. Core Flooding Experiment

**Figure 5:**
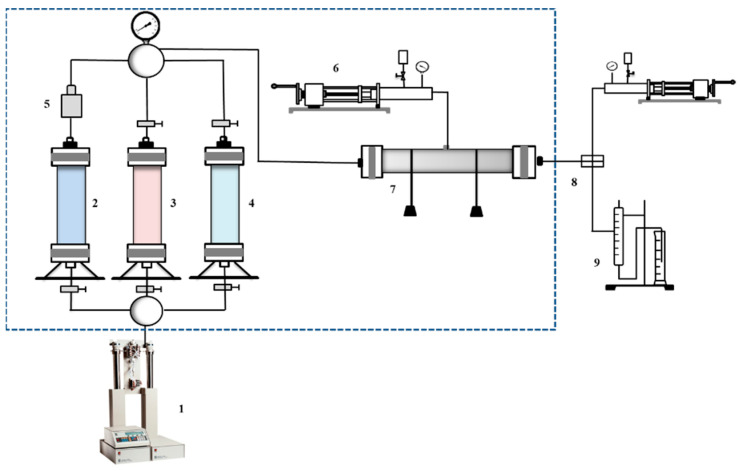
Flowchart of the experimental foam drive in two-pipe parallel core. 1—ISCO high-pressure piston pump; 2-4—Intermediate vessel; 5—One-way valve; 6–Hand pump; 7—Core gripper; 8—Return valve; 9—Gas-liquid separator.

In order to further investigate the transport law and blocking ability of CO_2_ nanobubble systems in a porous medium, we conducted an indoor long-core oil repulsion experiment to compare the recovery rate, production gas–oil ratio, and the dynamic change law of the differential pressure of repulsion in CO_2_ nanobubble systems using different repulsion methods. The timing of CO_2_ gas flushing and the control of different driving methods at the leading edge of driving were determined. The experimental flow of the double-tube parallel mandrel foam drive is shown in Figure 5. The oil-driving equipment utilized primarily included an ISCO X (ISCO Co., Lincoln, Nebraska, USA) double-liquid cylinder constant-pressure and constant-flow pump, an air pump (2XZ-2, Daxluot Instruments, Shanghai, China), a constant-temperature box, a surfactant solution and crude oil container, a foam viewing window, a gas flow controller, a pressure and temperature controller, and a core gripper. A sandstone core was selected, and the core parameters are shown in Table 1. The simulated oil viscosity was formulated to be 3.6 mPa·s, and the mineralization of the simulated formation water was 1970 mg/L. The ionic composition of the simulated formation water is shown in Table 2.

## 3. Results and Discussion

### 3.1. Structural Characterization of Modified Nanoparticles

**Figure 6:**
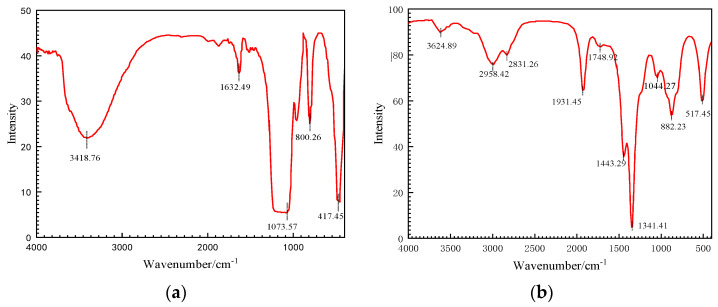
FTIR spectra of nano-SiO_2_ before and after modification: (**a**) FTIR spectra of SiO_2_ nanoparticles; (**b**) FTIR spectra of modified nano-SiO_2_.

In order to confirm the successful adsorption of MFDA on the surface of SiO_2_ particles, the MFDA-treated SiO_2_ particles were characterized via FTIR spectroscopy. The infrared spectra of untreated SiO_2_ particles and MFDA-modified SiO_2_ particles are shown in Figure 6. It can be observed that the FT-IR spectra of pure SiO_2_ and the FTIR spectra of surface-modified SiO_2_ demonstrate some differences in peak shape. As illustrated in Figure 6a, the anti-symmetric stretching vibration peak of structural water -OH is observed at a wave number of 3418.29 cm^−1^; in comparison, the H-O-H bending vibration peak is observed at a wave number of 162 cm^−1^. The bending vibration peak belonging to Si-OH is observed at 1073.57 cm^−1^; in comparison, the Si-O bond stretching vibration peak is observed at 417.45 cm^−1^. Figure 6b depicts the modified SiO_2_ with the -OH stretching vibration peak at 3624.89 cm^−1^. The CH_3_-bond stretching vibration peaks at 2958.42 cm^−1^ and 2831.26 cm^−1^, and the C=O stretching vibration peaks at 1931.4 cm^−1^. The stretching vibration peak induced by the presence of the C=O-O bond is observed at 1748.92 cm^−1^; in comparison, the O-H telescopic vibration peaks are seen at 1443.29 cm^−1^ and 1341.41 cm^−1^. The vibrational absorption peaks of C-H are observed at 882.23 cm^−1^. The symmetrical telescopic peaks of Si-O-CH_3_ were also generated, indicating that a certain amount of organic components exists on the surface of the modified nano-SiO_2_. The above findings prove that the organic and inorganic components were bonded to each other by the copolymerization and the condensation of -OH and that the organic and inorganic components were combined. The analysis results indicate that the modified nano-SiO_2_ is effectively formed.

**Figure 7:**
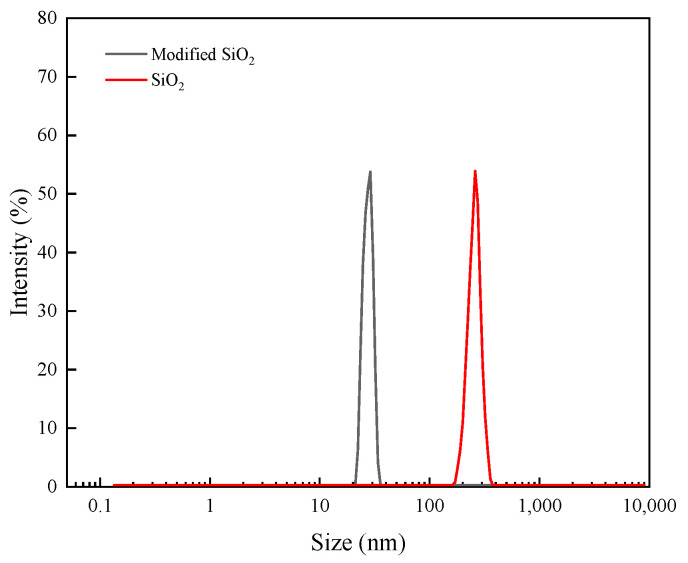
Distribution of nano-SiO_2_ particle sizes before and after modification.

In addition, a solution of 0.05 wt%silica nanoparticles was formulated in order to determine the hydrodynamic size distribution of the nanoparticles, as shown in Figure 7. The size distribution of the SiO_2_ nanoparticles before modification was determined to be between 126 nm and 356 nm. The peak of the distribution was observed to be 215 nm. The modified nano-SiO_2_ particle size ranged from 18 nm to 38 nm, with a peak of 25 nm. The above finding is due to the hydrophilic nature of unmodified nano-SiO_2_ particles, which are susceptible to agglomeration. This process results in an increase in particle size and the replacement of a portion of the hydroxyl group after modification, which reduces the agglomeration effect of the nano-SiO_2_ particles. This process facilitates the dispersion stability of SiO_2_ nanoparticles in aqueous solutions.

**Figure 8:**
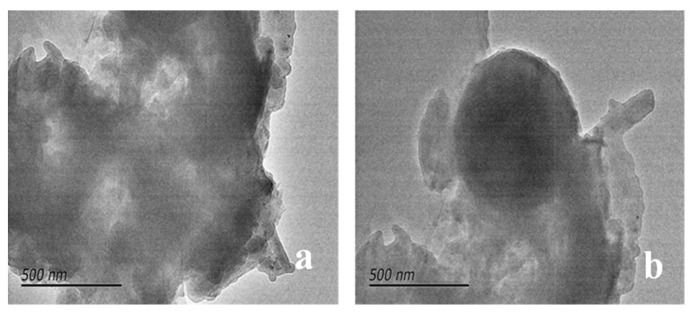
Scanning electron micrograph of nano-SiO_2_ before and after modification: (**a**) nano-SiO_2_ nanoparticles; (**b**) modified nano-SiO_2_.

Prior to the utilization of the nanoparticles, transmission electron microscopy (TEM) analysis was conducted to ascertain the dimensions and homogeneity of the nanoparticles both before and after modification. The results of the TEM analyses are presented in Figure 8. The SEM images demonstrate that the modified nanoparticles possess a nucleus of nano-SiO_2_, with external organic functional groups surrounding them. The modified nanoparticles exhibit enhanced dispersion characteristics, and the external hydrophobic functional groups reduce inter-particle agglomeration to a certain extent.

**Figure 9:**
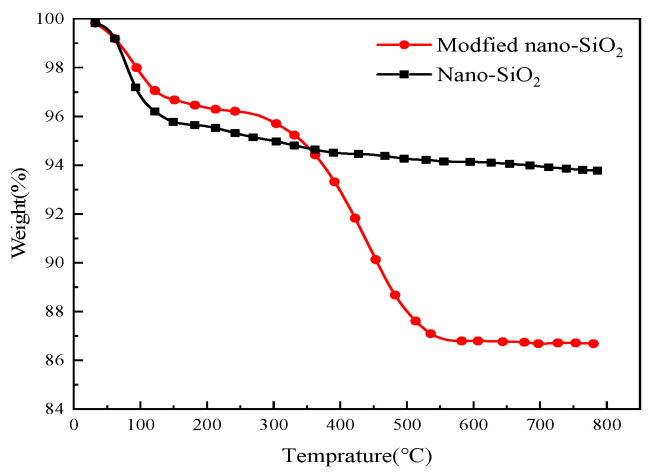
Thermo gravimetric analysis diagram.

The thermodynamic behavior of nano-SiO_2_ before and after modification was studied. As shown in Figure 9, the modified nano-silica particles exhibit two different weight-loss stages. The first stage took place at 100–300 °C, with a weight loss of 3.6%, which was due to the presence of free water and impurities on the surface of the nano-silica particles. The second stage took place at 400–800 °C, with a weight loss of 9.2%, possibly due to the decomposition of the MFDA polymer when heated. Conversely, their introduction helped improve the hydrophilic properties of the nano-silica particles, thereby reducing the mass of adsorbed water molecules and resulting in a decrease in the weight of the modified nano-silica particles.

### 3.2. Formation and Characterization of CO_2_ Nanobubbles

**Figure 10:**
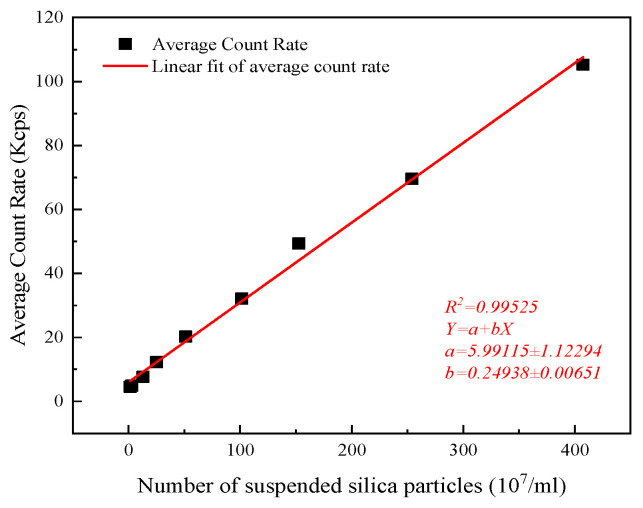
The relationship between the average count rate obtained from 90 plus PLAS and the number of suspended silica particles in Milli-Q water.

**Figure 11:**
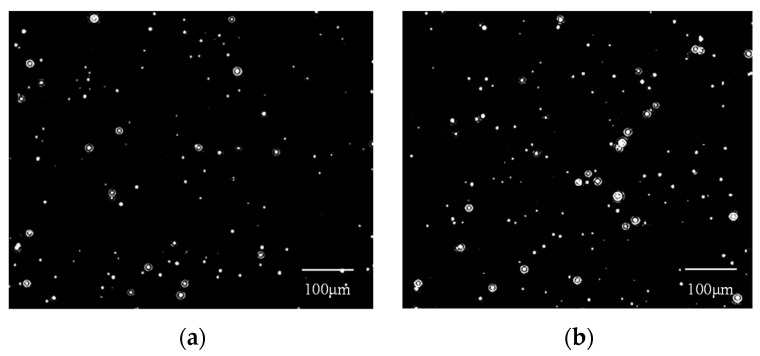
Images of CO_2_ nanobubbles obtained from NTA at different times: (**a**) 10 min; (**b**) 20 min.

The ACR values obtained when measuring nanobubbles using the DLS method can be employed to quantify the number concentration of nanobubbles. As illustrated in Figure 10, the ACR and the number of SiO_2_ nanoparticle suspensions are positively correlated. Consequently, the ACR value can be employed to quantify the number concentration of CO_2_ nanobubbles in water. The images of carbon dioxide nanobubbles obtained from NTA at different times are shown in Figure 11. A comparison of the nanobubble number concentration obtained using the NTA method with that obtained using the DLS quantitative method shows that a CO_2_ concentration of approximately 9.4 × 10^8^ particles/mL corresponds to an ACR value in the range of 30–35 Kcps. Quantifying the number concentration of CO_2_ nanobubbles in water can help determine the ideal technology and formulation for generating stable gas nanobubbles in distilled water [42]. Exploring the relationship between nanobubble size and stability will allow the matching of extra-low-permeability storage pore throat sizes for better applicability in the CO_2_-EOR field once the number concentration is known.

### 3.3. Surfactant Effect on CO_2_ Nanobubble System

**Figure 12:**
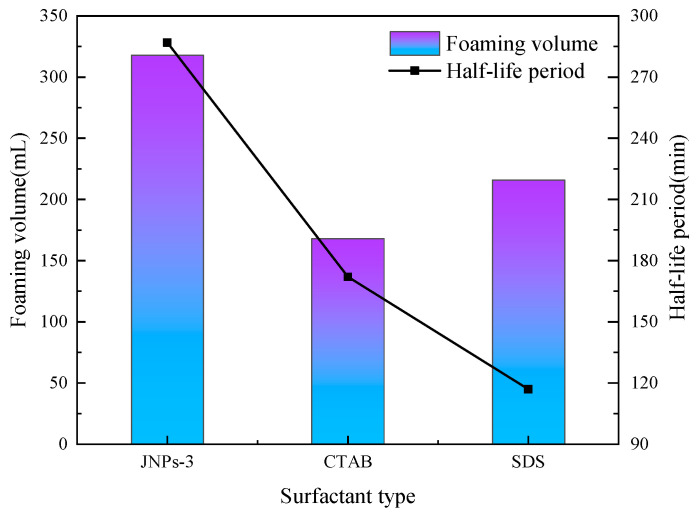
Effect of different surfactant types on foaming performance of CO_2_ nanobubble system.

In order to analyze the effect of surfactant type on the CO_2_ nanobubble system, three different types of surfactants with a concentration of 0.15 wt.% were selected and compounded with 0.05 wt.% nanoparticles to prepare a CO_2_ nanobubble system. The experimental results are shown in Figure 12. The foam performance of the system was evaluated, and it was found that JNPs-3 exhibited the most optimal foam performance, with a foam volume of 323 mL and a half-life of 327 min. This is because the particles are uniformly dispersed on the liquid film, and compared to when the particle dosage is high, the size and number of aggregates are significantly reduced. The liquid film has high mechanical strength and a low gravity effect to ensure the foam remains stable for a long time. At the same time, it can be found that the foam performance of the anionic–nonionic surfactant is higher than that of the cationic surfactant [43]. However, further validation is needed to determine whether the foaming performance of all anionic surfactants is superior to that of cationic surfactants. This experiment optimized the types and formulations of surfactants for CO_2_ nanobubble systems. Subsequently, a systematic performance evaluation was conducted on the formula.

### 3.4. Effect of Temperature on the Properties of CO_2_ Nanobubble System

**Figure 13:**
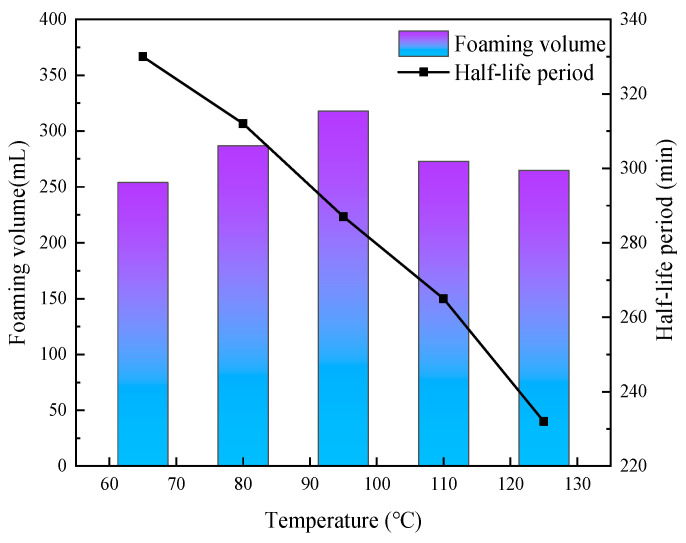
Effect of different temperatures on the foaming performance of CO_2_ nanobubble system.

**Figure 14:**
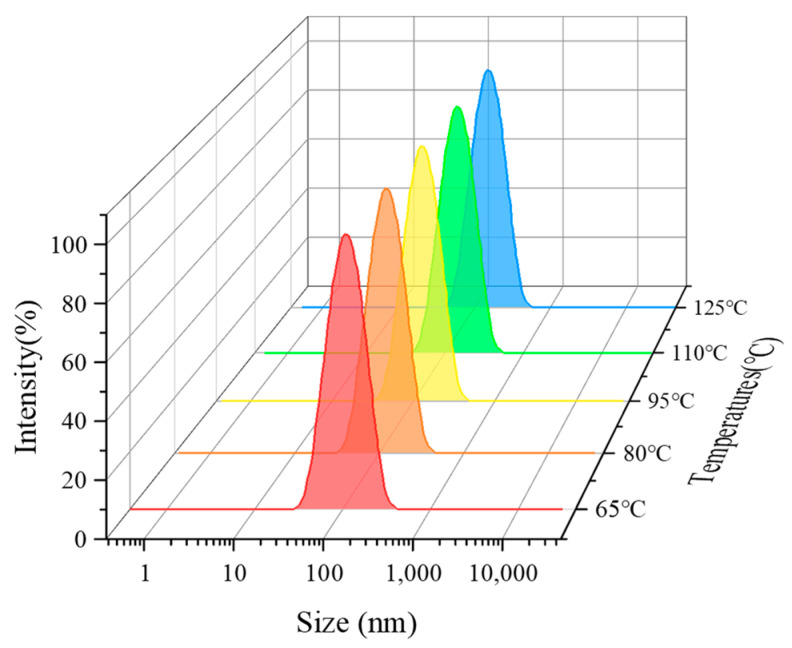
Effect of different temperatures on size of CO_2_ nanobubble system.

In order to study the effect of temperature on the CO_2_ nanobubble system, the foaming properties of the system were determined under different temperature conditions. As shown in Figure 13, the system still has excellent foaming performance at a temperature of 125 °C, with a foaming volume of 265 mL and a half-life of 232 min. In addition, it was found that temperature has a relatively small effect on bubble size, and the bubble size of the system can still be maintained at the nanometer level at higher temperatures, as shown in Figure 14. It is proved that the addition of nanoparticle foam stabilizer helps to improve the stability of the CO_2_ nanobubble system to a certain extent. As nano materials have certain temperature resistance characteristics, uniform adsorption and the gas–liquid interface can increase the foam performance of the CO_2_ nanobubble system under high-temperature conditions.

### 3.5. Effect of Oil Content on the Properties of CO_2_ Nanobubble System

**Figure 15:**
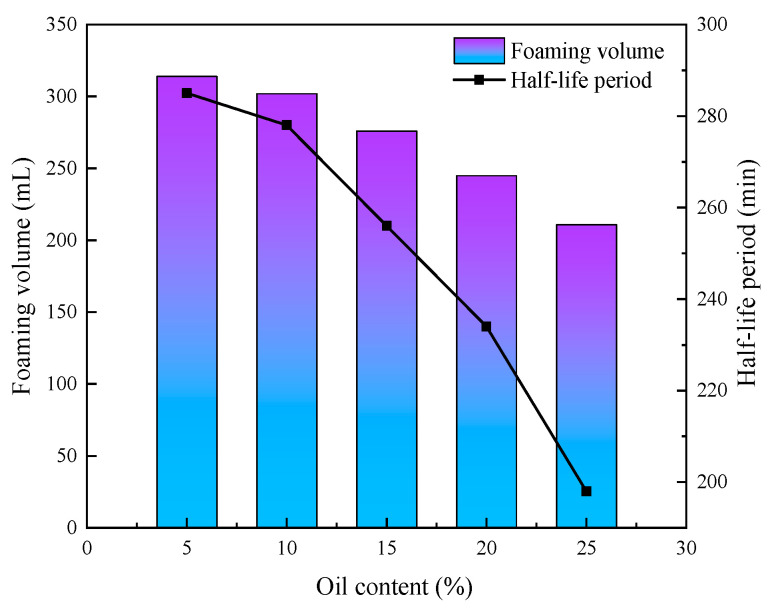
Effect of oil content on the foaming performance of CO_2_ nanobubble system.

**Figure 16:**
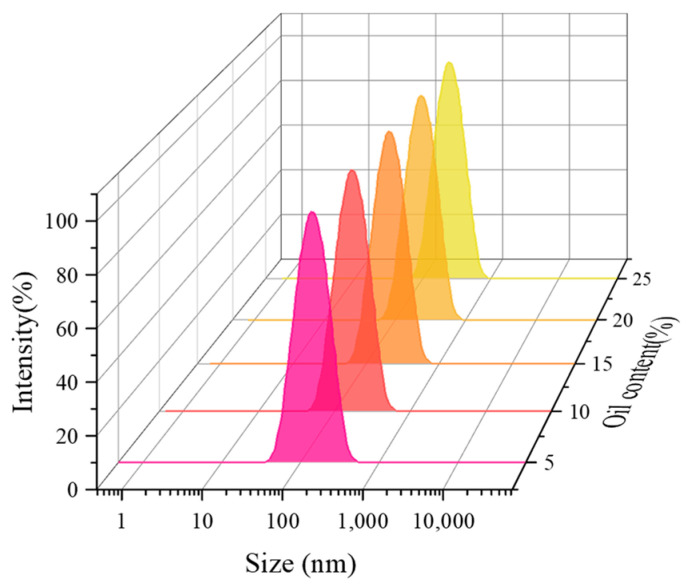
Effect of oil content on size of CO_2_ nanobubble system.

In order to study the effect of oil content on the CO_2_ nanobubble system, the foaming performance of the system was measured under different oil content conditions, and the experimental results show that the system has certain oil resistance properties. The experimental results are shown in Figure 15, where the foaming volume reaches 211 mL, with a half-life of 198 min at a 25% oil content. As shown in Figure 16, the oil content also has a small effect on the bubble size, and the bubble size can still be maintained in the nanometer range as the oil content increases. Crude oil has a great influence on the foaming ability and stability of foam agents. The foaming ability and foam agent with good stability measured by the Roche foam method become very unstable and easy to burst after contact with oil. This is because the combination of oil and active agent molecules participates in the arrangement of active agent molecules on the nanobubble membrane wall, resulting in changes in the stability and self-healing ability of the nanobubble liquid film, which reduces the stability of nanobubbles. However, the oil presence has no discernible negative impact on the rheology of Smart Nano-29-stabilised CO_2_ nanobubbles, which exhibit nearly identical maximum apparent viscosity and transitional foam quality values [44].

### 3.6. Contact Angle of CO_2_ Nanobubble System

**Figure 17:**
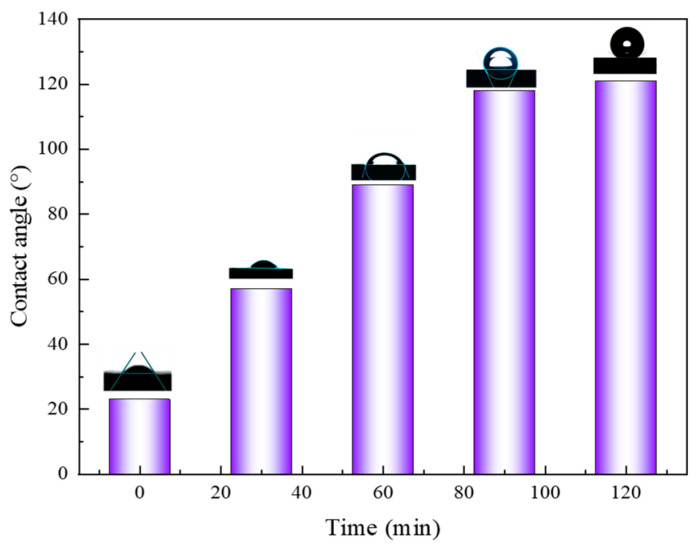
Contact angle variation with time for CO_2_ nanobubble systems.

In order to study the effect of the CO_2_ nanobubble system on the surface properties of the core, the contact angle of oil on the core slices of the target block was measured using a sessile drop method. The contact angle of oil was found to be 23° in the core slices that had been aged with crude oil, which demonstrates that the core slices at this time exhibited a certain degree of lipophilicity and were easily wetted by oil. Figure 17 illustrates the change in contact angle over time following immersion in the CO_2_ nanobubble system. As shown in Figure 17, the CO_2_ nanobubble system has a significant effect on the wettability of the core. It can be observed that the contact angle gradually increases with time, reaching a maximum value of 121° at 120 min. At this point, the core exhibits hydrophilic properties, indicating that the system is capable of facilitating the removal of crude oil by modifying the wettability of the rock.

### 3.7. Interfacial Tension of CO_2_ Nanobubble Systems

**Figure 18:**
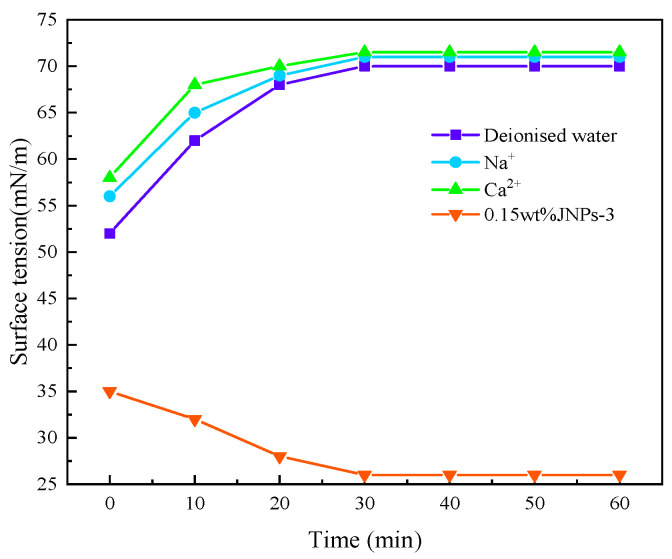
Interfacial tension of CO_2_ nanobubble systems.

The variation in surface tension is an important factor affecting the formation and stabilization of CO_2_ nanobubbles. In this study, the variation in the surface tension values of CO_2_ nanobubbles in different solutions was tested. The surface tension values of CO_2_ nanobubble systems formed by deionized water, Na^+^ solution, Ca^2+^ solution, and 0.15 wt%JNPs-3 were measured from 0 to 60 min in turn. The experimental results are shown in Figure 18. The surface tension value of CO_2_ nanobubbles formed using deionized water exhibited an initial value of 55 mN/m, with a gradual increase over time. This value reached 72 mN/m at around 40 min, which is comparable to the surface tension value of deionized water. This result is more consistent with the findings of Bu et al. [45]. While the surface tension of CO_2_ nanobubbles formed by Na^+^ and Ca^2+^ increased slightly, the surface tension value of the bubbles was close to that of deionized water at approximately 30 min. Conversely, the surface tension values of the nanobubbles formed by the surfactant JNPs-3 exhibited a gradual decrease; in comparison, those of CO_2_ nanobubbles with oil-containing surfactants exhibited only slight changes from 0 to 60 min. The above finding indicates that the enrichment of charged ions on the surface of the nanobubbles is sufficient to reduce the surface tension, thereby ensuring that the internal pressure of the bubbles is equal to the gas injection pressure. Consequently, the charge enrichment on the surface of the nanoscale bubbles and the lower surface tension of the supersaturated solution in comparison to that of the saturated solution are significant factors contributing to the equilibrium of the nanobubbles. A comparable outcome was observed in the calculations conducted by Ulatowski et al. in their study [42].

### 3.8. Core Flooding Experiment

**Figure 19:**
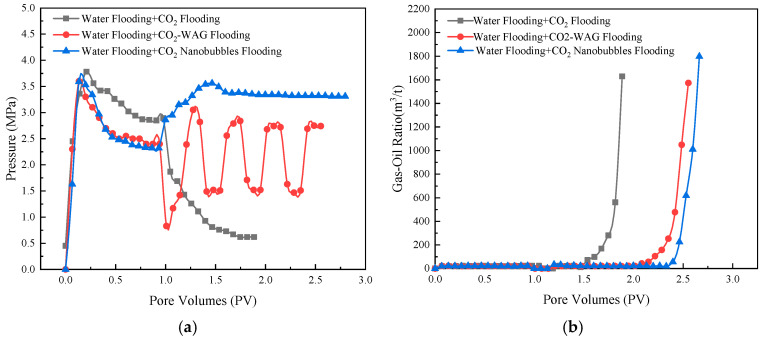
Variation in pressure and gas–oil ratio in different injection systems: (**a**) Variation in pressure; (**b**) Variation in gas–oil ratio.

Conclusions can be drawn from the analysis of the pressure angle, and the curves of the change in injection volume with pressure and the gas–liquid ratio are shown in Figure 19. The injection pressure of the three systems shows different trends with the change in injection volume. Following the replacement of the gas drive with the water drive, the pressure difference decreased markedly. The change rule of the water-alternating-gas flooding (WAG) stage after the water drive shows a fluctuating state, in which the pressure decreases during the same round of gas injection and increases during liquid injection. Following the water drive, the pressure rises rapidly to 3.56 MPa at the commencement of injection, subsequently stabilizing at 0.66 MPa of the CO_2_ nanobubble drive before finally stabilizing at 3.31 MPa.

It can be observed that changes in the gas–liquid ratio are also indicative of a delay in the gas–liquid ratio during the rising stage. This phenomenon is in comparison to the continuous gas drive and the gas–liquid alternating drive, where the CO_2_ nanobubble drive was delayed by 0.97 PV and 0.39 PV, respectively. These results indicate that the CO_2_ nanobubble system has a certain degree of tampering prevention effect. This phenomenon can be attributed to the high gas density and lateral diffusivity of the nanobubbles. As the contraction of the nanobubbles reaches its limit, the air pressure inside the bubbles tends toward infinity. This self-pressurization effect results in the rupture of the nanobubble–water interface, thereby facilitating the efficient transfer of gas from the nanobubbles to the surrounding liquid.

**Figure 20:**
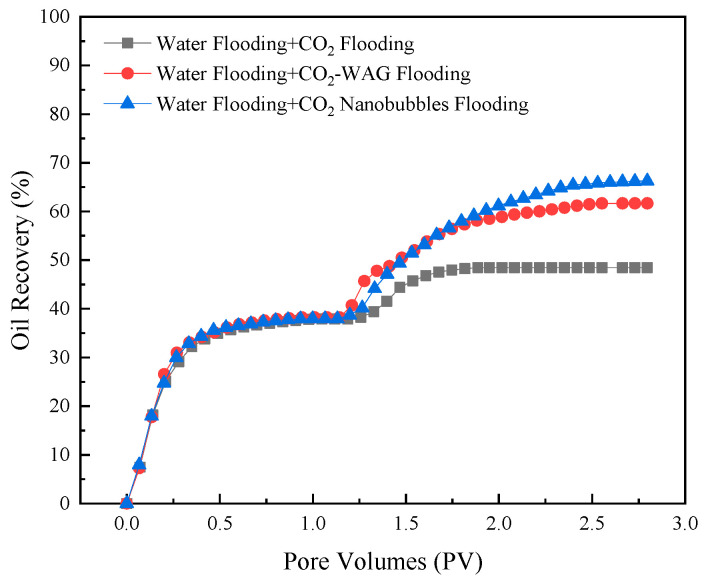
Variation in recovery rates for different injection systems.

In order to evaluate the recovery enhancement ability of the CO_2_ nanobubble system, a one-dimensional linear experiment utilizing natural core splicing was simulated in the oil-driving experiment. The CO_2_ nanobubble system was formulated as 0.15 wt%JNPs-3 + 0.05 wt%Smart Nano-29. Figure 20 illustrates the recovery outcomes of the core experiments conducted with distinct driving methodologies. The experimental outcomes demonstrate that the recovery rate of CO_2_ flooding subsequent to water flooding is 48.64%; in comparison, that of WAG flooding subsequent to water flooding is 61.65%. These findings demonstrate that the recovery rate can be enhanced by 13.01% in comparison to simple water flooding and CO_2_ flooding. The recovery rate of the CO_2_ nanobubble drive after water flooding is 66.28%, which demonstrates that the recovery rate can be enhanced by 17.64% in comparison to simple water flooding + CO_2_ flooding. The injection of a CO_2_ nanobubble system into the system allows for the control of the leading edge of the drive to a certain extent. This factor results in a greater differential pressure within the drive, which, in turn, forces the injected gas to enter the previously unreached area. This process effectively replaces the remaining crude oil within the core, thereby further enhancing the recovery of oil and gas [46].

## 4. Conclusions

In this work, we evaluate the effectiveness of CO_2_ nanobubble systems for application in ultra-low-permeability reservoirs. A modified nano-SiO_2_ particle with pro-CO_2_ properties was designed using the Pickering emulsion template method and employed as a CO_2_ nanobubble stabilizer. A CO_2_-philic hydrophobic polymer monomer was used to stabilize the size of the nanobubbles via electrostatic adsorption and hydrogen bonding while preventing CO_2_ spillage in the system from destabilizing the nanobubble system. This process also prevents the CO_2_ overflow in the system from destroying the stability of the nano-foam system and is used as a foam stabilizer in the CO_2_ nano-foam system. The CO_2_ nanobubbles exhibited excellent temperature resistance, oil resistance, dimensional stability, interfacial properties, and wetting-reversal properties. The EOR effect of the CO_2_ nanobubble systems was evaluated by core experiments, resulting in a core simulation experiment in which the recovery rate can reach 66.28%. The injected CO_2_ is readily gas-scrambled, which reduces the exposure time and contact area of CO_2_ and the extra-low-permeability reservoir. This makes it difficult to effectively play the role of CO_2_ molecular diffusion, resulting in a large amount of residual oil stagnation. Through the study of the performance of CO_2_ nanobubble systems, it has been found that they can play a certain blocking role, effectively reducing the occurrence of carbon dioxide channeling. Unlike the traditional carbon dioxide foam, the CO_2_ nanobubble systems have certain advantages, such as small size, and can play a better role in ultra-low-permeability reservoirs. The findings in this paper can provide researchers with insights into future research directions and recommendations for CO_2_-EOR.

The uncertainty that may affect the results in the core oil drive experiments mainly comes from the instability of the CO_2_ high-pressure gas itself, and the phase changes are greatly affected by temperature and pressure. Subsequently, we will address the flow pattern of CO_2_ nanobubbles in porous media.

## Figures and Tables

**Table 1:** Core parameters and experimental program.

Core Number	Core Size	Permeability (10^−3^ μm^2^)	Experimental Program
L-1	99.82	21.67	Water flooding to 98% water content + CO_2_ flooding to 1500 m^3^/t gas/oil ratio end
L-2	98.65	24.19	Water flooding to 98% water content + CO_2_/water flooding to 1500 m^3^/t gas/oil ratio end
L-3	99.73	22.36	Water flooding to 98% water content + CO_2_ nanobubbles to 1500 m^3^/t gas/oil ratio end

**Table 2:** Modeling the ionic composition of formation water (mg/L).

Na^+^	K^+^	Ca^2+^	Mg^2+^	HCO^3−^	Cl^−^
581.85	10.49	22.75	8.04	898.38	448.38

## Data Availability

Restrictions apply to the datasets. The datasets presented in this paper are not readily available due to the fact that the research fund project is in the ongoing research phase, and the data are part of the ongoing research. Requests to access the datasets should be directed to sygc9212@163.com.
